# Association of caffeine intake with all-cause and cardiovascular mortality in elderly patients with hypertension

**DOI:** 10.3389/fnut.2022.1023345

**Published:** 2022-12-20

**Authors:** Shuaijie Chen, Jing Li, Menghan Gao, Duanbin Li, Ruming Shen, Lingchun Lyu, Jiayi Shen, Xiaohua Shen, Guosheng Fu, Tiemin Wei, Wenbin Zhang

**Affiliations:** ^1^Department of Cardiology, Sir Run Run Shaw Hospital, College of Medicine, Zhejiang University, Hangzhou, China; ^2^Department of Cardiology, Lishui Hospital, College of Medicine, Zhejiang University, Lishui, China; ^3^College of Medicine, Zhejiang University, Hangzhou, China

**Keywords:** caffeine intake, mortality, cardiovascular risk, hypertension, elderly patients

## Abstract

**Background:**

Caffeine is widely consumed not only in coffee but also in soft drinks and tea. However, the long-term health effects of caffeine are still controversial, especially in people with high cardiovascular risk such as elderly patients with hypertension.

**Methods:**

This study analyzed data from the National Health and Nutrition Examination Survey 2003–2018. Caffeine intake was calculated by two 24-h dietary recall interviews. Complex sampling-weighted multivariable Cox proportional hazards models were used to compare the hazard ratios (HRs) of all-cause and cardiovascular mortality in elderly hypertensive patients with different caffeine intake (<10, 10 to <100, 100 to <200, 200 to <300, and ≥300 mg/day).

**Results:**

This study included 6,076 elderly hypertensive patients. The mean ± standard error follow-up duration was 6.86 ± 0.12 years. During this period, a total of 2,200 all-cause deaths occurred, of which 765 were cardiovascular deaths. Taking patients with caffeine intake < 10 mg/day as a reference, patients with moderate caffeine intake (200 to <300 mg/day) had a lower risk of all-cause (HR, 0.70 [95% CI, 0.56–0.87]) and cardiovascular (HR, 0.55 [95% CI, 0.39–0.77]) mortality. The benefit of reducing all-cause mortality risk was significant in female patients (HR, 0.65 [95% CI, 0.50–0.85]) or patients with well-controlled blood pressure (HR, 0.63 [95% CI, 0.46–0.87]), but not in male patients or patients with poorly controlled blood pressure. In addition, non-linear relationship analysis also showed that moderate caffeine intake had the lowest HRs of all-cause (Non-linear *p* = 0.022) and cardiovascular mortality (Non-linear *p* = 0.032) in the present study.

**Conclusion:**

Moderate caffeine intake is associated with reduced risk of all-cause and cardiovascular mortality in elderly hypertensive patients.

## Introduction

Coffee is one of the most widely consumed beverages in the world. Numerous observational studies have shown that moderate coffee consumption has beneficial effects on the circulatory system, central nervous system, respiratory system, immune system, etc. (1–6). And present research found an inverse relationship between moderate coffee consumption with all-cause and cause-specific mortality (7–10).

Caffeine is consumed not only in coffee but also in soft drinks and tea. And because coffee also contains many other ingredients, caffeine and coffee cannot be considered the same. The relationship between caffeine intake and cardiovascular disease risk remains controversial. Excessive intake of caffeine can lead to increases in sympathetic nerve activity and circulating catecholamine concentrations, mediated by stimulation of the central nervous system (11, 12). Caffeine is thought to acutely increase blood pressure in caffeine-sensitive individuals, which may temporarily increase the risk of cardiovascular events (1, 6, 13). This acute effect limits the recommendation of coffee and caffeine in the hypertensive population, although there is no evidence that long-term habitual caffeine intake is associated with hypertension and cardiovascular risk. Currently, studies investigating the association between caffeine intake and mortality remain sparse, especially in hypertensive patients. Elderly hypertensive patients are at high risk of cardiovascular death and may be more susceptible to caffeine intake (14–17). Therefore, this study aimed to assess the association of caffeine intake with all-cause and cardiovascular mortality in elderly hypertensive patients.

## Materials and methods

### Study participants

All information on participants in this retrospective cohort study was obtained from the National Health and Nutrition Examination Survey (NHANES) (18). The NHANES is a program of studies designed to assess the health and nutritional status of the population in the US. This survey is unique in that it combines interviews and physical examinations (18). The US National Center for Health Statistics (NCHS) has the responsibility for producing vital health statistics, using a stratified, multi-stage probability sampling design that enables participants to be representative of the civilian deinstitutionalized population of the US.

**FIGURE 1:**
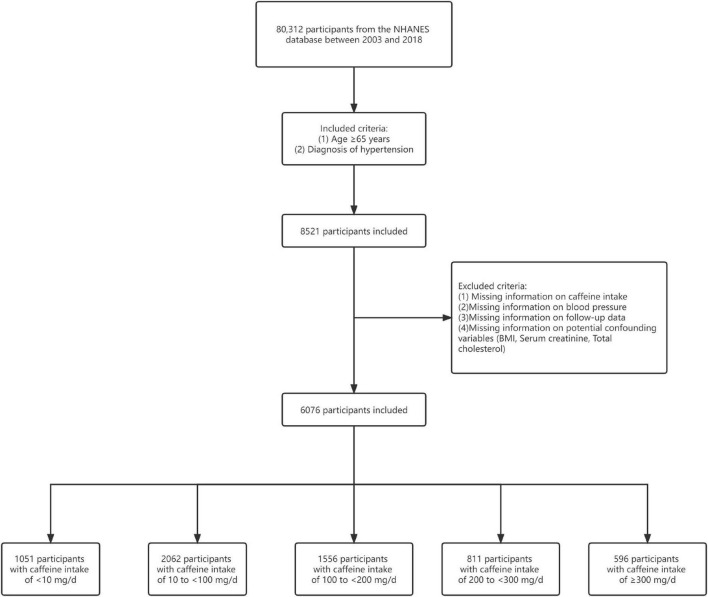
Flowchart of patient selection.

This study obtained data on 80,312 participants between 2003 and 2018 from the NHANES database. We focused on participants aged ≥65 years with hypertension (*n* = 8,521). Hypertension was defined as measured systolic blood pressure (SBP) ≥140 mmHg or/and diastolic blood pressure (DBP) ≥90 mmHg, or/and previous diagnosis of hypertension, or/and taking antihypertensive prescription. Of these, we excluded patients with missing information on caffeine intake (*n* = 1,780). Then, patients with missing blood pressure data were also excluded (*n* = 180). Additionally, participants lacking follow-up data were excluded (*n* = 8). Finally, we excluded those patients with missing information on potential confounding variables (*n* = 477). A total of 6,076 participants were included for analysis in this study (Figure 1). The follow-up period is from the date of participation in the survey until December 31, 2019 (19). The NCHS Research Ethics Review Board approved the NHANES protocol (20). The NHANES has obtained written informed consent from each participant.

### Caffeine intake assessment

Since 2003, all NHANES participants are eligible for two 24-h dietary recall interviews. The first dietary recall interview is collected in person in the Mobile Examination Center and the second interview is collected by telephone 3–10 days later (21). The dietary intake data are used to estimate the types and amounts of foods and beverages (including all types of water) consumed during the 24-h period prior to the interview (midnight to midnight). Then, The NHANES used the US Department of Agriculture Food and Nutrient Database for Dietary Studies (FNDDS) (22) to identify those foods and beverages as intakes of various nutrients and food ingredients. The FNDDS contained data on the nutritional content of all foods and beverages consumed by participants, including more than 7,000 individual foods and beverages. The exact caffeine content of various foods and drinks was also available on the FNDDS (23). Sources of caffeine include coffee, tea, soda, energy drinks, milk, other beverages and foods, and versions with or without caffeine were considered (24). The study divided participants into five groups based on their 2-day average caffeine intake (<10, 10 to <100, 100 to <200, 200 to <300, and ≥300 mg/day).

### Outcome ascertainment

The outcomes of this study were all-cause and cardiovascular death. All-cause death was defined as death from any cause. According to the International Classification of Diseases, 10th revision (ICD-10), cardiovascular death was defined as death due to I00-I09, I11, I13, I20-I51, and I60-I69. The NHANES linked mortality data have been updated with mortality follow-up data through December 31, 2019 (19).

### Covariate assessment

The following variables were obtained through the interview questionnaire: age, sex, race/ethnicity, educational level, smoking status (current, former, and never), medical conditions (including hypertension, diabetes, asthma, heart failure, coronary heart disease, stroke, emphysema, thyroid problem, chronic bronchitis, liver condition, and cancer), and antihypertensive medication use. Total daily energy, protein, carbohydrate, sugar, dietary fiber, fat, alcohol, and sodium intakes were obtained in the same way as the caffeine intake assessment above. The following variables were measured according to standard protocols: body mass index (BMI; calculated as weight in kilograms divided by height in meters squared), blood pressure, total cholesterol, triglycerides, high density lipoprotein, serum creatinine, and uric acid. The estimated glomerular filtration rate (eGFR) was calculated by the Chronic Kidney Disease Epidemiology Collaboration equation (25). Hyperuricemia was defined as serum uric acid level >420 mcmol/L for male patients and >360 mcmol/L for female patients. Obese was defined as BMI ≥ 30 kg/m^2^. Well-controlled blood pressure was defined as SBP < 140 mmHg and DBP < 90 mmHg.

### Statistical analysis

The NHANES samples are not simple random samples, and complex survey designs need to be considered. Following the recommendations of the US Centers for Disease Control and Prevention for the analysis of NHANES data (26), we used appropriate weights for each analysis based on the selected variables. Weights are provided by NCHS. Continuous variables were expressed as mean ± standard error (mean ± SE), the normality of each continuous variable was evaluated using Kolmogorov Smirnov test, the comparison between groups of continuous variables with normal distribution using analysis of variance, and the comparison between groups of continuous variables with non-normal distribution using Kruskal–Wallis test. Categorical variables were expressed as percentages, and comparisons between groups were made by the chi-square test. For the analysis of mortality outcomes, multivariable Cox proportional hazards regression was performed to calculate hazard ratios (HR) and 95% confidence intervals (CI) with reference to participants with caffeine intake <10 mg/day. Model 1 adjusted for age, sex, race/ethnicity, educational level, smoking status, and BMI. Adjustments for model 2 included adjustments for model 1 plus non-cardiovascular disease conditions (diabetes, asthma, emphysema, thyroid problem, chronic bronchitis, liver condition, and cancer), eGFR, uric acid, total daily energy, protein, carbohydrate, sugar, dietary fiber, fat, alcohol, and sodium intakes. Adjustments for model 3 included adjustments for model 2 plus cardiovascular disease conditions (heart failure, coronary heart disease, and stroke), SBP, DBP, total cholesterol, triglycerides, and high-density lipoprotein. The HRs for the most adequately adjusted model 3 were analyzed separately by sex, blood pressure control, hyperuricemia, obese, or eGFR. Restricted cubic spline (RCS) regression with three knots at the 10th, 50th, and 90th centiles was used to explore the non-linear relationship between caffeine intake and mortality. All statistical analyses were performed using R version 4.1.2 (R Project for Statistical Computing). *P* < 0.05 was regarded as statistically significant for all tests.

## Results

### Baseline characteristics

Table 1 summarizes the weighted baseline information for 6,076 elderly hypertensive patients. Among total patients, the mean ± SE age was 73.39 ± 0.12 years, 43% were male, 81.2% were self-identified as non-Hispanic white, and the mean ± SE caffeine intake was 146.16 ± 2.98 mg/day. Compared with patients with lower caffeine intake, patients with higher caffeine intake were younger, more likely to be male, non-Hispanic white, and smoker, had higher education level and intake of various nutrients, higher eGFR level, lower systolic blood pressure, lower high density lipoprotein, and lower prevalence of asthma. Notably, although not statistically significant, patients with higher caffeine intake may be predisposed to coronary heart disease. And compared with other groups, the 200 to <300 group may have a higher prevalence of heart failure and cancer, and a lower prevalence of stroke.

**TABLE 1:** Participants’ baseline demographic and clinical characteristics.

Characteristic	Total	Caffeine intake (mg/day)					*P*-value
		**<10**	**10 to <100**	**100 to <200**	**200 to <300**	**≥300**	
Unweighted sample (*n*)	6,076	1,051	2,062	1,556	811	596	
Age (years)	73.39 ± 0.12	73.76 ± 0.29	74.16 ± 0.18	73.41 ± 0.23	72.79 ± 0.28	71.77 ± 0.24	<0.001
Sex (%)							<0.001
Male	43	37.9	36.2	39.2	53.1	61.2	
Female	57	62.1	63.8	60.8	46.9	38.8	
Race (%)							<0.001
Mexican American	3.7	3.2	5	3.6	2.9	2.3	
Other Hispanic	2.8	2.8	3.9	2.9	1.6	1.1	
Non-Hispanic white	81.2	74.3	73.3	83.8	90.8	90.9	
Non-Hispanic black	8.3	15.4	12.1	6.2	3	1.4	
Other race	4	4.3	5.7	3.5	1.8	4.3	
Education level (%)							<0.001
Less than high school	21.7	24.4	25.8	20.6	15.7	19	
At least high school	78.3	75.6	74.2	79.4	84.3	81	
Smoking status (%)							<0.001
Current	7.4	4.5	5.4	6.5	7.8	18.2	
Former	43.5	40.1	38.9	40.2	54.3	52.5	
Never	49	55.4	55.7	53.3	38	29.3	
Body mass index (kg/m^2^)	29.31 ± 0.11	29.23 ± 0.28	29.06 ± 0.19	29.13 ± 0.25	29.95 ± 0.34	29.53 ± 0.30	0.109
Body mass index (%)							0.067
<30	60.6	60.1	62.5	63.1	57.4	55.1	
≥30	39.4	39.9	37.5	36.9	42.6	44.9	
Systolic blood pressure (mmHg)	138.81 ± 0.41	138.40 ± 0.96	141.15 ± 0.73	137.91 ± 0.82	137.75 ± 0.96	137.24 ± 1.28	0.011
Diastolic blood pressure (mmHg)	66.10 ± 0.30	65.99 ± 0.77	65.52 ± 0.46	66.03 ± 0.56	66.44 ± 0.72	67.33 ± 0.94	0.396
Total cholesterol (mmol/L)	4.97 ± 0.03	5.01 ± 0.07	5.00 ± 0.04	4.95 ± 0.04	4.93 ± 0.05	4.93 ± 0.08	0.629
Triglycerides (mmol/L)	1.77 ± 0.02	1.65 ± 0.05	1.81 ± 0.04	1.79 ± 0.05	1.76 ± 0.04	1.78 ± 0.07	0.110
High density lipoprotein (mmol/L)	1.44 ± 0.01	1.48 ± 0.03	1.42 ± 0.01	1.48 ± 0.02	1.40 ± 0.02	1.37 ± 0.02	0.001
Uric acid (mcmol/L)	344.69 ± 1.69	339.90 ± 5.11	343.94 ± 2.88	341.49 ± 3.21	354.99 ± 4.90	346.08 ± 4.79	0.158
Hyperuricemia (%)	28.5	28.3	30.3	26.6	30.1	26.5	0.514
eGFR (ml/min/1.73 m^2^)	67.89 ± 0.35	68.49 ± 0.89	66.11 ± 0.54	67.68 ± 0.62	68.51 ± 0.90	71.14 ± 0.89	<0.001
eGFR (%)							0.019
<60	32.5	32.6	35.2	34.6	30.7	22.9	
≥60	67.5	67.2	64.7	66.6	68.5	75.5	
Energy (kcal)	1,780.59 ± 13.58	1,645.53 ± 29.90	1,673.13 ± 19.30	1,747.23 ± 21.53	1,959.58 ± 30.06	2,056.60 ± 40.00	<0.001
Protein (gm)	69.73 ± 0.58	66.46 ± 1.56	65.80 ± 0.83	67.16 ± 0.90	76.07 ± 1.18	81.04 ± 0.80	<0.001
Carbohydrate (gm)	215.51 ± 1.58	203.63 ± 3.85	207.65 ± 2.33	213.88 ± 2.86	228.89 ± 3.91	236.11 ± 6.11	<0.001
Total sugar (gm)	94.30 ± 0.87	88.94 ± 2.15	93.32 ± 1.33	93.72 ± 1.76	98.93 ± 2.43	98.86 ± 3.12	0.044
Dietary fiber (gm)	16.33 ± 0.16	16.31 ± 0.37	15.73 ± 0.26	16.10 ± 0.27	16.93 ± 0.29	17.58 ± 0.50	0.001
Total fat (gm)	69.89 ± 0.66	62.42 ± 1.42	64.45 ± 1.01	68.10 ± 1.05	79.33 ± 1.57	84.47 ± 2.02	<0.001
Alcohol (gm)	5.69 ± 0.30	4.74 ± 0.74	4.15 ± 0.37	5.57 ± 0.51	7.79 ± 0.89	8.10 ± 1.01	<0.001
Sodium (mg)	2,957.24 ± 26.53	2,732.39 ± 59.52	2,752.45 ± 37.87	2,878.13 ± 42.53	3,290.50 ± 60.08	3,486.76 ± 82.34	<0.001
Diabetes (%)	23.1	20.5	23	23.1	25	23.6	0.678
Asthma (%)	12.8	17.6	14	12	10	8.9	0.005
Heart failure (%)	8.6	9	9.7	6.6	11.2	6.5	0.015
Coronary heart disease (%)	13.4	13.4	12.4	12.1	14.4	17.9	0.105
Stroke (%)	9.7	9.8	12	8.5	7.4	9.6	0.043
Emphysema (%)	4.7	3.9	4.7	3.8	4.8	7.8	0.038
Thyroid problem (%)	22.2	23.7	22.1	24.4	21.2	16.6	0.118
Chronic bronchitis (%)	8.7	9.7	8	6.9	11.1	10.2	0.077
Liver condition (%)	4.4	3.1	5.2	4	5.2	4	0.459
Cancer (%)	28.2	30.1	24.6	28.8	32.7	26.4	0.05

eGFR, estimated glomerular filtration rate. Continuous variables are expressed as means and standard error and categorical variables as percentages. Means and percentages are weighted.

### Association of caffeine intake with all-cause and cardiovascular mortality

During the follow-up of 6,076 participants, a total of 2,200 all-cause deaths occurred, of which 765 were cardiovascular deaths. The mean ± SE follow-up duration was 6.86 ± 0.12 years. The results of Cox regression analysis were shown in Table 2.

**TABLE 2:** Hazard ratios for all-cause and cardiovascular mortality of all participants, stratified by caffeine intake.

Outcomes	Caffeine intake (mg/day)				
	**<10**	**10 to <100**	**100 to <200**	**200 to <300**	**≥300**
**All-cause mortality**					
Unadjusted HR	1 [Ref]	**0.84 (0.72–0.99)**	0.86 (0.72–1.02)	**0.76 (0.61–0.95)**	**0.75 (0.59–0.96)**
*P*		**0.035**	0.081	**0.014**	**0.024**
Model 1 HR	1 [Ref]	0.87 (0.74–1.01)	0.89 (0.75–1.06)	**0.79 (0.63–0.99)**	0.83 (0.64–1.07)
*P*		0.076	0.189	**0.040**	0.147
Model 2 HR	1 [Ref]	**0.80 (0.69–0.93)**	**0.83 (0.70–0.98)**	**0.73 (0.58–0.91)**	**0.77 (0.60–1.00)**
*P*		**0.004**	**0.027**	**0.005**	**0.047**
Model 3 HR	1 [Ref]	**0.77 (0.66–0.90)**	**0.83 (0.70–0.98)**	**0.70 (0.56–0.87)**	**0.74 (0.57–0.96)**
*P*		**<0.001**	**0.026**	**0.001**	**0.023**
**Cardiovascular mortality**					
Unadjusted HR	1 [Ref]	0.82 (0.61–1.11)	0.89 (0.67–1.17)	**0.63 (0.45–0.89)**	0.75 (0.51–1.09)
*P*		0.206	0.402	**0.008**	0.134
Model 1 HR	1 [Ref]	0.85 (0.63–1.16)	0.93 (0.70–1.24)	**0.68 (0.48–0.97)**	0.90 (0.60–1.36)
*P*		0.315	0.607	**0.035**	0.624
Model 2 HR	1 [Ref]	0.76 (0.57–1.01)	0.84 (0.63–1.10)	**0.61 (0.43–0.88)**	0.85 (0.57–1.26)
*P*		0.056	0.203	**0.008**	0.408
Model 3 HR	1 [Ref]	**0.69 (0.52–0.91)**	0.80 (0.61–1.06)	**0.55 (0.39–0.77)**	0.76 (0.51–1.14)
*P*		**0.009**	0.12	**<0.001**	0.184

HR, Hazard ratio; Ref, reference; BMI, body mass index; eGFR, estimated glomerular filtration rate; SBP, systolic blood pressure; DBP, diastolic blood pressure. Model 1: adjusted for age, sex, race/ethnicity, educational level, smoking status, and BMI. Model 2: adjustments for model 1 plus non-cardiovascular disease conditions (diabetes, asthma, emphysema, thyroid problem, chronic bronchitis, liver condition, and cancer), eGFR, uric acid, total daily energy, protein, carbohydrate, sugar, dietary fiber, fat, alcohol, and sodium intakes. Model 3: adjustments for model 2 plus cardiovascular disease conditions (heart failure, coronary heart disease, and stroke), SBP, DBP, total cholesterol, triglycerides, and high-density lipoprotein. Statistically significant HR and *p*-values were shown in bold.

The most adequately adjusted model 3 results show that moderate caffeine intake can reduce all-cause and cardiovascular mortality in elderly hypertensive patients. Taking caffeine intake <10 mg/day as reference, the risk of all-cause mortality was significantly reduced in all groups. Patients with caffeine intake of 200 to <300 mg/day had the lowest HR of all-cause mortality (HR, 0.70 [95% CI, 0.56–0.87]). For cardiovascular mortality, we observed similar results. Taking caffeine intake <10 mg/day as a reference, the full-adjusted HR for cardiovascular mortality in patients with caffeine intake of 200 to <300 mg/day was 0.55 [95% CI, 0.39–0.77].

The results of parameter estimation in cox models were listed in Supplementary materials. In addition to caffeine intake, age, diabetes, emphysema, systolic and diastolic blood pressure, heart failure, and stroke were all associated with all-cause and cardiovascular mortality.

### Subgroup analysis

**FIGURE 2:**
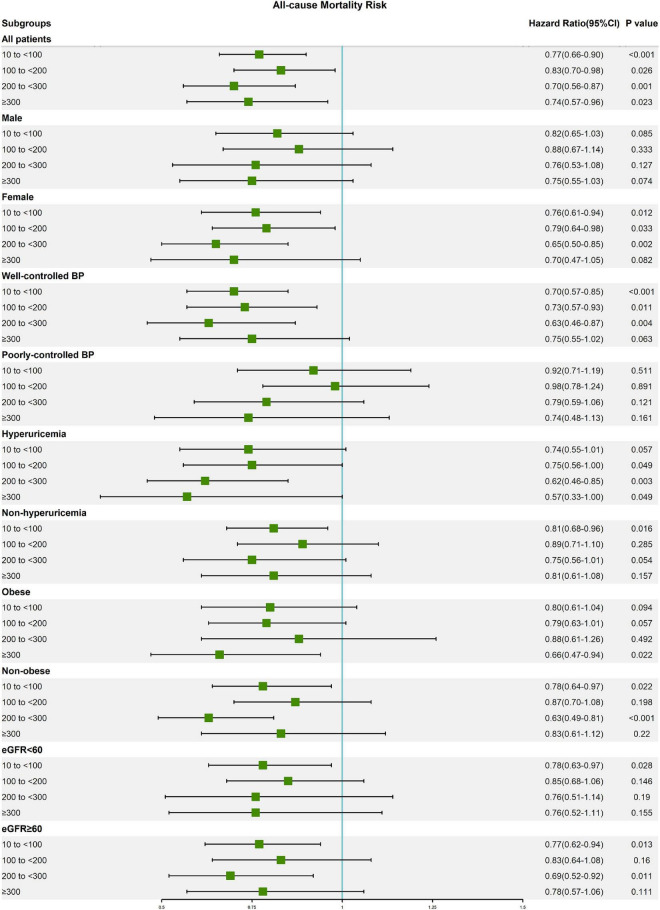
Forest plot of multivariable model 3 hazard ratios for all-cause mortality in subgroup analysis.

**FIGURE 3:**
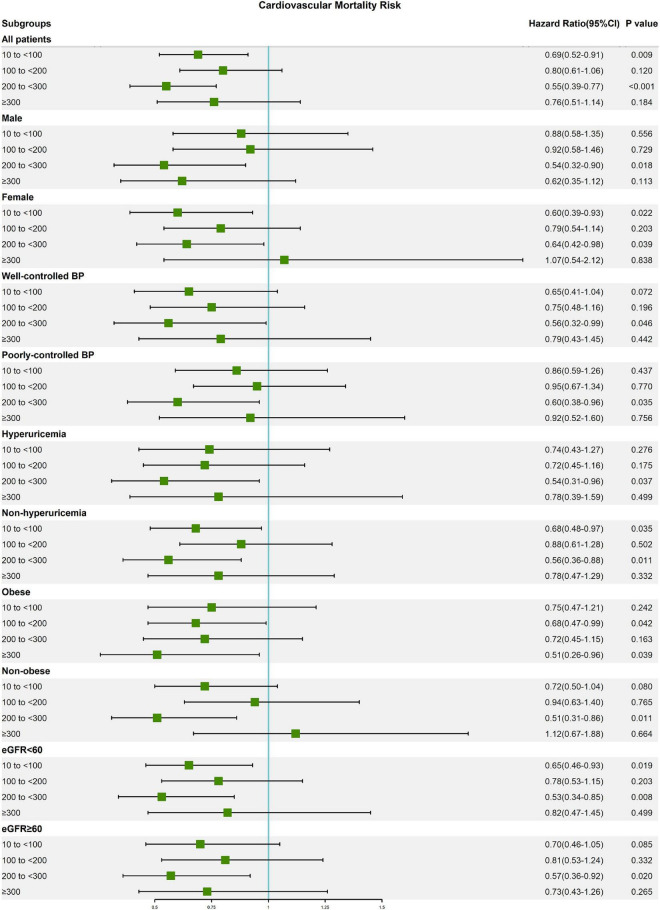
Forest plot of multivariable model 3 hazard ratios for cardiovascular mortality in subgroup analysis.

The results of subgroup analysis according to the most adequately adjusted Cox regression model 3 are presented in Table 3. Except for the male subgroup and poorly controlled blood pressure subgroup, caffeine intake was beneficial in reducing all-cause and cardiovascular mortality of elderly hypertensive patients in all subgroups. And no significant interaction was observed between each subgroup (Sex, Blood pressure control, Hyperuricemia, Obese, and eGFR) and caffeine intake groups (Supplementary Table 1). For all-cause mortality (Figure 2), caffeine intake showed a significant benefit in the female subgroup (10 to <100 mg/day: HR, 0.76 [95% CI, 0.61–0.94]; 100 to <200 mg/day: HR, 0.79 [95% CI, 0.64–0.98]; and 200 to <300 mg/day: HR, 0.65 [95% CI, 0.50–0.85] compared with female participants with caffeine intake of <10 mg/day) and well-controlled blood pressure subgroup (10 to <100 mg/day: HR, 0.70 [95% CI, 0.57–0.85]; 100 to <200 mg/day: HR, 0.73 [95% CI, 0.57–0.93]; and 200 to <300 mg/day: HR, 0.63 [95% CI, 0.46–0.87] compared with well-controlled blood pressure participants with caffeine intake of <10 mg/day), but not in the male subgroup and poorly controlled blood pressure subgroup. In addition, the hyperuricemia subgroup had lower HRs for all-cause mortality than the non-hyperuricemia subgroup. Interestingly, caffeine intake with the lowest HR of all-cause mortality is different in obese subgroup (≥300 mg/day: HR, 0.66 [95% CI, 0.47–0.94]) and non-obese subgroup (200 to <300 mg/day: HR, 0.63 [95% CI, 0.49–0.81]). For cardiovascular mortality (Figure 3), moderate caffeine intake (200 to <300 mg/day) showed a significant benefit in all subgroups, except for the obese subgroup. In the obese subgroup, caffeine intake with the lowest HR of cardiovascular mortality was ≥300 mg/day (HR, 0.51 [95% CI, 0.26–0.96]), which was similar to the results of all-cause mortality. We also observed that caffeine intake of 10 to <100 mg/day significantly reduced cardiovascular mortality risk in the female subgroup (HR, 0.60 [95% CI, 0.39–0.93]).

**TABLE 3:** Multivariable model 3 hazard ratios for all-cause and cardiovascular mortality of subgroups (Sex, Blood pressure control, Hyperuricemia, Obese, and eGFR), stratified by caffeine intake.

Outcomes	Death/n	Caffeine intake (mg/day)				
		**<10**	**10 to <100**	**100 to <200**	**200 to <300**	**≥300**
**All-cause mortality**						
**Sex**						
Male	1,162/2,910	1 [Ref]	0.82 (0.65–1.03)	0.88 (0.67–1.14)	0.76 (0.53–1.08)	0.75 (0.55–1.03)
*P*			0.085	0.333	0.127	0.074
Female	1,038/3,166	1 [Ref]	**0.76 (0.61–0.94)**	**0.79 (0.64–0.98)**	**0.65 (0.50–0.85)**	0.70 (0.47–1.05)
*P*			**0.012**	**0.033**	**0.002**	0.082
**Blood pressure control**						
Yes	1,074/3,133	1 [Ref]	**0.70 (0.57–0.85)**	**0.73 (0.57–0.93)**	**0.63 (0.46–0.87)**	0.75 (0.55–1.02)
*P*			**<0.001**	**0.011**	**0.004**	0.063
No	1,126/2,943	1 [Ref]	0.92 (0.71–1.19)	0.98 (0.78–1.24)	0.79 (0.59–1.06)	0.74 (0.48–1.13)
*P*			0.511	0.891	0.121	0.161
**Hyperuricemia**						
Yes	723/1,750	1 [Ref]	0.74 (0.55–1.01)	**0.75 (0.56–1.00)**	**0.62 (0.46–0.85)**	**0.57 (0.33–1.00)**
*P*			0.057	**0.049**	**0.003**	**0.049**
No	1,477/4,326	1 [Ref]	**0.81 (0.68–0.96)**	0.89 (0.71–1.10)	0.75 (0.56–1.01)	0.81 (0.61–1.08)
*P*			**0.016**	0.285	0.054	0.157
**Obese**						
Yes	1,445/3,743	1 [Ref]	0.80 (0.61–1.04)	0.79 (0.63–1.01)	0.88 (0.61–1.26)	**0.66 (0.47–0.94)**
*P*			0.094	0.057	0.492	**0.022**
No	755/2,333	1 [Ref]	**0.78 (0.64–0.97)**	0.87 (0.70–1.08)	**0.63 (0.49–0.81)**	0.83 (0.61–1.12)
*P*			**0.022**	0.198	**<0.001**	0.22
**eGFR**						
<60	1,011/2,020	1 [Ref]	**0.78 (0.63–0.97)**	0.85 (0.68–1.06)	0.76 (0.51–1.14)	0.76 (0.52–1.11)
*P*			**0.028**	0.146	0.19	0.155
≥60	1,189/4,056	1 [Ref]	**0.77 (0.62–0.94)**	0.83 (0.64–1.08)	**0.69 (0.52–0.92)**	0.78 (0.57–1.06)
*P*			**0.013**	0.16	**0.011**	0.111
**Cardiovascular mortality**						
**Sex**						
Male	409/2,910	1 [Ref]	0.88 (0.58–1.35)	0.92 (0.58–1.46)	**0.54 (0.32–0.90)**	0.62 (0.35–1.12)
*P*			0.556	0.729	**0.018**	0.113
Female	356/3,166	1 [Ref]	**0.60 (0.39–0.93)**	0.79 (0.54–1.14)	**0.64 (0.42–0.98)**	1.07 (0.54–2.12)
*P*			**0.022**	0.203	**0.039**	0.838
**Blood pressure control**						
Yes	366/3,133	1 [Ref]	0.65 (0.41–1.04)	0.75 (0.48–1.16)	**0.56 (0.32–0.99)**	0.79 (0.43–1.45)
*P*			0.072	0.196	**0.046**	0.442
No	399/2,943	1 [Ref]	0.86 (0.59–1.26)	0.95 (0.67–1.34)	**0.60 (0.38–0.96)**	0.92 (0.52–1.60)
*P*			0.437	0.77	**0.035**	0.756
**Hyperuricemia**						
Yes	266/1,750	1 [Ref]	0.74 (0.43–1.27)	0.72 (0.45–1.16)	**0.54 (0.31–0.96)**	0.78 (0.39–1.59)
*P*			0.276	0.175	**0.037**	0.499
No	499/4,326	1 [Ref]	**0.68 (0.48–0.97)**	0.88 (0.61–1.28)	**0.56 (0.36–0.88)**	0.78 (0.47–1.29)
*P*			**0.035**	0.502	**0.011**	0.332
**Obese**						
Yes	484/3,743	1 [Ref]	0.75 (0.47–1.21)	**0.68 (0.47–0.99)**	0.72 (0.45–1.15)	**0.51 (0.26–0.96)**
*P*			0.242	**0.042**	0.163	**0.039**
No	281/2,333	1 [Ref]	0.72 (0.50–1.04)	0.94 (0.63–1.40)	**0.51 (0.31–0.86)**	1.12 (0.67–1.88)
*P*			0.08	0.765	**0.011**	0.664
**eGFR**						
<60	378/2,020	1 [Ref]	**0.65 (0.46–0.93)**	0.78 (0.53–1.15)	**0.53 (0.34–0.85)**	0.82 (0.47–1.45)
*P*			**0.019**	0.203	**0.008**	0.499
≥60	387/4,056	1 [Ref]	0.70 (0.46–1.05)	0.81 (0.53–1.24)	**0.57 (0.36–0.92)**	0.73 (0.43–1.26)
*P*			0.085	0.332	**0.02**	0.265

HR, Hazard ratio; Ref, reference; BMI, body mass index; eGFR, estimated glomerular filtration rate; SBP, systolic blood pressure; DBP, diastolic blood pressure. Model 3: adjustments for age, sex, race/ethnicity, educational level, smoking status, BMI, non-cardiovascular disease conditions (diabetes, asthma, emphysema, thyroid problem, chronic bronchitis, liver condition, and cancer), eGFR, uric acid, energy intake, protein intake, carbohydrate intake, sugar intake, dietary fiber intake, fat intake, alcohol intake, sodium intake, cardiovascular disease conditions (heart failure, coronary heart disease, and stroke), SBP, DBP, total cholesterol, triglycerides, and high-density lipoprotein. Statistically significant HR and *p*-values were shown in bold.

### Non-linear relationship analysis

**FIGURE 4:**
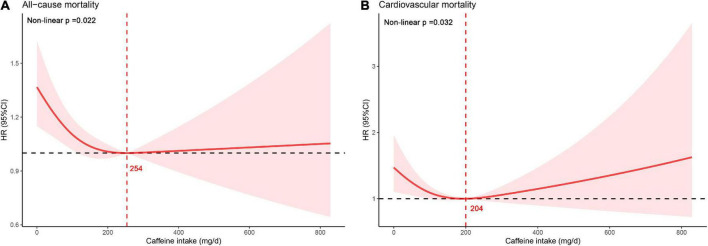
Non-linear relationship between caffeine intake and mortality. **(A)** All-cause mortality. **(B)** Cardiovascular mortality.

The most adequately adjusted RCS regression results are shown in Figure 4. There was a significant non-linear relationship between caffeine intake and all-cause mortality in elderly hypertensive patients (Non-linear *p* = 0.022), and caffeine intake with the lowest HR was 254 mg/day. There was also a significant non-linear relationship between caffeine intake and cardiovascular mortality in elderly hypertensive patients (Non-linear *p* = 0.032), and caffeine intake with the lowest HR was 204 mg/day. Non-linear relationship analysis also showed that moderate caffeine intake can reduce all-cause and cardiovascular mortality in elderly hypertensive patients.

### Sensitivity analysis

Sensitivity analyses were performed to test the robustness of the results, including removing participants with extreme energy intake (energy intake <1,000 kcal or energy intake >3,000 kcal) or with baseline history of cardiovascular diseases and cancer or with missing low density lipoprotein data (low density lipoprotein included in the model). The results of three sensitivity analyses based on model 3 were basically consistent with the above results (Table 4). Participants with moderate caffeine intake (200 to <300 mg/day) had significantly lower HRs for all-cause and cardiovascular mortality compared with participants with caffeine intake of <10 mg/day.

**TABLE 4:** Sensitivity analysis between caffeine intake with mortality based on multivariable model 3.

Outcomes	Death/n	Caffeine intake (mg/day)				
		**<10**	**10 to <100**	**100 to <200**	**200 to <300**	**≥300**
**All-cause mortality**						
Remove participants with extreme energy intake	1,901/5,236	1 [Ref]	**0.77 (0.66–0.90)**	**0.81 (0.67–0.99)**	**0.71 (0.57–0.88)**	0.80 (0.61–1.04)
*P*			**<0.001**	**0.035**	**0.002**	0.099
Remove participants with baseline history of CVD and cancer	967/3,280	1 [Ref]	0.80 (0.63–1.01)	0.87 (0.69–1.12)	**0.63 (0.47–0.85)**	0.82 (0.56–1.21)
*P*			0.06	0.283	**0.003**	0.318
Low density lipoprotein included	1,049/2,876	1 [Ref]	**0.75 (0.59–0.94)**	**0.75 (0.60–0.92)**	**0.55 (0.44–0.70)**	**0.64 (0.44–0.92)**
*P*			**0.014**	**0.007**	**<0.001**	**0.016**
**Cardiovascular mortality**						
Remove participants with extreme energy intake	649/5,236	1 [Ref]	**0.68 (0.52–0.90)**	0.74 (0.52–1.04)	**0.54 (0.37–0.79)**	0.75 (0.48–1.18)
*P*			**0.007**	0.084	**0.002**	0.217
Remove participants with baseline history of CVD and cancer	313/3,280	1 [Ref]	0.67 (0.43–1.04)	0.82 (0.57–1.18)	**0.46 (0.29–0.73)**	0.68 (0.40–1.15)
*P*			0.076	0.289	**0.001**	0.146
Low density lipoprotein included	376/2,876	1 [Ref]	0.76 (0.51–1.12)	0.79 (0.55–1.14)	**0.41 (0.23–0.72)**	0.93 (0.53–1.63)
*P*			0.167	0.213	**0.002**	0.805

HR, Hazard ratio; Ref, reference; BMI, body mass index; eGFR, estimated glomerular filtration rate; SBP, systolic blood pressure; DBP, diastolic blood pressure; CVD, Cardiovascular diseases. Model 3: adjustments for age, sex, race/ethnicity, educational level, smoking status, BMI, non-cardiovascular disease conditions (diabetes, asthma, emphysema, thyroid problem, chronic bronchitis, liver condition and cancer), eGFR, uric acid, energy intake, protein intake, carbohydrate intake, sugar intake, dietary fiber intake, fat intake, alcohol intake, sodium intake, cardiovascular disease conditions (heart failure, coronary heart disease and stroke), SBP, DBP, total cholesterol, triglycerides, and high-density lipoprotein. Extreme energy intake was defined as energy intake <1,000 kcal or energy intake >3,000 kcal. Cardiovascular diseases were defined as coronary heart disease, heart failure, stroke, angina, and heart attack. Model with low density lipoprotein included: adjustments for model 3 plus low density lipoprotein. Statistically significant HR and *p*-values were shown in bold.

## Discussion

In this study, we observed that moderate caffeine intake was associated with decreased risk of all-cause and cardiovascular mortality in elderly hypertensive patients. The HRs for all-cause and cardiovascular mortality were lowest in participants with caffeine intake of 200 to <300 mg/day (one cup of coffee contains approximately 100 mg of caffeine). Although no significant interaction was observed, elderly hypertensive female patients or patients with well-controlled blood pressure may be more likely to benefit from caffeine intake, further studies are warranted to elucidate differences in the effects of caffeine intake among various populations.

Many studies have demonstrated a beneficial effect of moderate coffee consumption on the risk of all-cause and cardiovascular mortality (1, 8, 27–29). But the effects of caffeine and coffee cannot be viewed on the same level. Caffeine, as a most important ingredient in coffee, has received extensive attention. Several studies in recent years have also observed that caffeine reduces the risk of all-cause mortality (9, 10, 30). However, the relationship between caffeine and cardiovascular mortality is still controversial. Feng et al. (30) suggested that higher caffeine intake was associated with lower cardiovascular mortality. A study on an elderly population also supports this view (14). But several studies have shown no association between caffeine intake and cardiovascular mortality (9, 10). In addition, a study suggests that caffeine consumption in patients with cardiovascular disease may increase the risk of cardiovascular death (7). In this regard, the effect of caffeine on blood pressure may limit its reduction in the risk of cardiovascular death. Past studies have found that caffeine induces an acute vasopressor effect that increases blood pressure (6, 14). Coffee consumption was associated with uncontrolled blood pressure in a hypertensive elderly population (15). At present, research on the long-term effects of caffeine in hypertensive patients is still limited, especially in the elderly population with high cardiovascular risk.

As early as 1988, Martin et al. (31) proposed that increased levels of caffeine consumption were not associated with increased all-cause or cardiovascular mortality in hypertensive patients aged 30–69 years over the subsequent 4 years. However, follow-up time was insufficient, and covariates were not fully adjusted in their study. In addition, with the dramatic changes in dietary caffeine, results from decades ago are no longer appropriate for the current situation. Recently, Palatini et al. (32) found that coffee is a dietary risk factor for adverse cardiovascular outcomes in hypertensive patients. But they mainly focused on coffee consumption without specifically assessing caffeine intake. Besides, their study was limited to Caucasian stage 1 hypertensive patients aged from 18 to 45 years and the proportion of outcome events was too small. Based on NHANES data from 1999 to 2010, Tsujimoto et al. (10) found that moderate caffeine intake can reduce the risk of all-cause mortality in hypertensive patients. However, Tsujimoto et al. (10) did not further assess the effect of caffeine intake on the risk of cardiovascular death in hypertensive patients.

Our study used 2003–2018 NHANES data and applied complex sampling weighted multivariable Cox proportional hazards models to assess the association of caffeine intake with cardiovascular and all-cause mortality in elderly patients with hypertension in detail. Furthermore, we performed subgroup analysis and performed RCS analysis to evaluate the specific non-linear relationship. Therefore, our study is novel and reliable. The present study suggests that appropriate caffeine intake is beneficial in reducing the risk of all-cause and cardiovascular mortality in elderly hypertensive patients. This extends the recommendation of moderate caffeine intake for elderly people with hypertension based on previous research, which is conducive to the long-term health management of elderly hypertensive patients.

Caffeine, as one of the most commonly consumed substances, can widely act on various systems of the human body, such as the circulatory system, central nervous system, respiratory system, immune system, etc. (17). The health benefits of caffeine may be due to its antioxidant and anti-inflammatory properties. About the circulatory system, it has been suggested that caffeine has a positive inotropic effect, possibly related to an increase in intracellular calcium concentration, release of norepinephrine, and sensitization of dopamine receptors (33). In general, acute caffeine intake stimulates a modest increase in blood pressure. However, long-term chronic caffeine intake did not show a blood pressure-raising effect (6). Interestingly, it has been suggested that caffeine metabolites may reduce the incidence of hypertension (34). Even considering the potential blood pressure-raising effect of caffeine, we believe that the benefits of caffeine in elderly hypertensive patients with well-controlled blood pressure are worthy of recognition.

This study found that the HRs of mortality in women were lower than that in men, especially all-cause mortality. the effect of caffeine intake on mortality may be potentially gender-differentiated, and several previous studies have reported similar results (10, 35). This may be related to genes, hormone levels, sensitivity to caffeine, etc. (36–38). Further studies are necessary to explore the underlying specific mechanisms. We also found that obesity may have affected the beneficial effects of caffeine intake, which is consistent with previous studies (9, 10, 30). This may be related to the tendency of obese patients to consume more caffeine-containing diets, which suggests that obese patients may be able to adapt to higher caffeine intake. More studies are needed to clarify the appropriate caffeine intake for people with different weight statuses. In addition, hyperuricemia is a risk factor for kidney disease and cardiovascular disease (16, 39–41). Some studies have suggested that caffeine intake and coffee consumption are inversely associated with serum uric acid levels (42–44). Our study observed that elderly hypertensive patients with hyperuricemia had lower HRs for all-cause mortality than those without hyperuricemia. This suggests that elderly hypertensive patients with hyperuricemia may be more inclined to obtain the benefits of caffeine, and further research is warranted to clarify the differences in the effect of caffeine intake in people with different uric acid levels. We considered that the benefits of caffeine may vary among elderly hypertensive patients with different statuses. Appropriate caffeine intake according to the condition of elderly hypertensive patients is healthy.

This is the first study of caffeine on the risk of death in elderly hypertensive patients. The sample size and follow-up time included in this study were sufficient. And we took full account of various potential confounding factors, including demographic variables, dietary intakes, laboratory measurements, medical conditions, etc. However, the research still has several limitations. First, caffeine intake was estimated based on self-reported dietary information, and there was inevitable measurement error. Second, 2-day average caffeine intake may not accurately reflect long-term caffeine intake. Last but not least, this study is a retrospective cohort study. Even after adjusting for many confounding variables, there is still a substantial risk of confounding bias.

## Conclusion

Moderate caffeine intake is associated with reduced risk of all-cause and cardiovascular mortality in elderly hypertensive patients. Although no significant interaction was observed, the benefits of caffeine may be more pronounced in elderly hypertensive female patients or patients with well-controlled blood pressure, and further studies are warranted to elucidate differences in the effects of caffeine intake among various populations.

## Data availability statement

Publicly available datasets were analyzed in this study. This data can be found here: NHANES Questionnaires, Datasets, and Related Documentation (https://wwwn.cdc.gov/nchs/nhanes/Default.aspx).

## Ethics statement

The studies involving human participants were reviewed and approved by National Center for Health Statistics Research Ethics Review Board approved the NHANES protocol (https://www.cdc.gov/nchs/nhanes/irba98.htm). The patients/participants provided their written informed consent to participate in this study.

## Author contributions

WZ and SC conceived and designed the research. JL, MG, DL, RS, and JS processed data and performed the statistical analysis. SC, JL, and MG wrote the initial manuscript. WZ, TW, GF, LL, and XS reviewed and corrected the manuscript. All authors read and approved the final manuscript.
