# Supplementary material for: Association of caffeine intake with all-cause and cardiovascular mortality in elderly patients with hypertension
Source: Front Nutr. 2022 Dec 20;9:1023345. doi: 10.3389/fnut.2022.1023345 (PMC9807616; doi:10.3389/fnut.2022.1023345)
Supplement: Supplementary file 2 [file Table_1.docx]

**Supplementary Table 1. Interaction analysis** **between each subgroup (Sex, Blood pressure control, Hyperuricemia, Obese, eGFR) and caffeine intake groups.**

| **Outcomes** | **Caffeine intake, mg/d** | | | | |
| --- | --- | --- | --- | --- | --- |
|  | **<10** | **10 to <100** | **100 to <200** | **200 to <300** | **≥300** |
| **All-cause mortality** | |  |  |  |  |
| **Sex** |  |  |  |  |  |
| Male | 1 [Ref] | 0.82(0.65-1.03) | 0.88(0.67-1.14) | 0.76(0.53-1.08) | 0.75(0.55-1.03) |
| Female | 1 [Ref] | **0.76(0.61-0.94)** | **0.79(0.64-0.98)** | **0.65(0.50-0.85)** | 0.70(0.47-1.05) |
| P for interaction |  | 0.716 | 0.479 | 0.475 | 0.42 |
| **Blood pressure control** |  |  |  |  |  |
| Yes | 1 [Ref] | **0.70(0.57-0.85)** | **0.73(0.57-0.93)** | **0.63(0.46-0.87)** | 0.75(0.55-1.02) |
| No | 1 [Ref] | 0.92(0.71-1.19) | 0.98(0.78-1.24) | 0.79(0.59-1.06) | 0.74(0.48-1.13) |
| P for interaction |  | 0.061 | 0.098 | 0.444 | 0.787 |
| **Hyperuricemia** |  |  |  |  |  |
| Yes | 1 [Ref] | 0.74(0.55-1.01) | **0.75(0.56-1.00)** | **0.62(0.46-0.85)** | **0.57(0.33-1.00)** |
| No | 1 [Ref] | **0.81(0.68-0.96)** | 0.89(0.71-1.10) | 0.75(0.56-1.01) | 0.81(0.61-1.08) |
| P for interaction |  | 0.625 | 0.425 | 0.197 | 0.076 |
| **Obese** |  |  |  |  |  |
| Yes | 1 [Ref] | 0.80(0.61-1.04) | 0.79(0.63-1.01) | 0.88(0.61-1.26) | **0.66(0.47-0.94)** |
| No | 1 [Ref] | **0.78(0.64-0.97)** | 0.87(0.70-1.08) | **0.63(0.49-0.81)** | 0.83(0.61-1.12) |
| P for interaction |  | 0.979 | 0.528 | 0.117 | 0.262 |
| **eGFR** |  |  |  |  |  |
| <60 | 1 [Ref] | **0.78(0.63-0.97)** | 0.85(0.68-1.06) | 0.76(0.51-1.14) | 0.76(0.52-1.11) |
| ≥60 | 1 [Ref] | **0.77(0.62-0.94)** | 0.83(0.64-1.08) | **0.69(0.52-0.92)** | 0.78(0.57-1.06) |
| P for interaction |  | 0.618 | 0.636 | 0.663 | 0.979 |
| **Cardiovascular mortality** | |  |  |  |  |
| **Sex** |  |  |  |  |  |
| Male | 1 [Ref] | 0.88(0.58-1.35) | 0.92(0.58-1.46) | **0.54(0.32-0.90)** | 0.62(0.35-1.12) |
| Female | 1 [Ref] | **0.60(0.39-0.93)** | 0.79(0.54-1.14) | **0.64(0.42-0.98)** | 1.07(0.54-2.12) |
| P for interaction |  | 0.361 | 0.582 | 0.716 | 0.436 |
| **Blood pressure control** |  |  |  |  |  |
| Yes | 1 [Ref] | 0.65(0.41-1.04) | 0.75(0.48-1.16) | **0.56(0.32-0.99)** | 0.79(0.43-1.45) |
| No | 1 [Ref] | 0.86(0.59-1.26) | 0.95(0.67-1.34) | **0.60(0.38-0.96)** | 0.92(0.52-1.60) |
| P for interaction |  | 0.566 | 0.623 | 0.816 | 0.987 |
| **Hyperuricemia** |  |  |  |  |  |
| Yes | 1 [Ref] | 0.74(0.43-1.27) | 0.72(0.45-1.16) | **0.54(0.31-0.96)** | 0.78(0.39-1.59) |
| No | 1 [Ref] | **0.68(0.48-0.97)** | 0.88(0.61-1.28) | **0.56(0.36-0.88)** | 0.78(0.47-1.29) |
| P for interaction |  | 0.876 | 0.693 | 0.784 | 0.759 |
| **Obese** |  |  |  |  |  |
| Yes | 1 [Ref] | 0.75(0.47-1.21) | **0.68(0.47-0.99)** | 0.72(0.45-1.15) | **0.51(0.26-0.96)** |
| No | 1 [Ref] | 0.72(0.50-1.04) | 0.94(0.63-1.40) | **0.51(0.31-0.86)** | 1.12(0.67-1.88) |
| P for interaction |  | 0.954 | 0.339 | 0.221 | 0.082 |
| **eGFR** |  |  |  |  |  |
| <60 | 1 [Ref] | **0.65(0.46-0.93)** | 0.78(0.53-1.15) | **0.53(0.34-0.85)** | 0.82(0.47-1.45) |
| ≥60 | 1 [Ref] | 0.70(0.46-1.05) | 0.81(0.53-1.24) | **0.57(0.36-0.92)** | 0.73(0.43-1.26) |
| P for interaction |  | 0.844 | 0.871 | 0.702 | 0.887 |
| HR, Hazard ratio; Ref, reference; BMI, body mass index; eGFR, estimated glomerular filtration rate; SBP, systolic blood pressure; DBP, diastolic blood pressure.  Model 3: adjustments for age, sex, race/ethnicity, educational level, smoking status, BMI, non-cardiovascular disease conditions (diabetes, asthma, emphysema, thyroid problem, chronic bronchitis, liver condition and cancer), eGFR, uric acid, energy intake, protein intake, carbohydrate intake, sugar intake, dietary fiber intake, fat intake, alcohol intake, sodium intake, cardiovascular disease conditions (heart failure, coronary heart disease and stroke), SBP, DBP, total cholesterol, triglycerides, and high-density lipoprotein.  Statistically significant HR and p-values were shown in bold. | | | | | |
